# Supplementary material for: Multi-omic profiling of clear cell renal cell carcinoma identifies metabolic reprogramming associated with disease progression
Source: Nat Genet. 2024 Feb 15;56(3):442–57. doi: 10.1038/s41588-024-01662-5 (PMC10937392; doi:10.1038/s41588-024-01662-5)
Supplement: Supplementary file 1 — Supplementary Figs. 1–5 and Supplementary Note. [file 41588_2024_1662_MOESM1_ESM.pdf]

nature genetics

Article https://doi.org/10.1038/s41588-024-01662-5

# Multi-omic profiling of clear cell renal cell carcinoma identifies metabolic reprogramming associated with disease progression

In the format provided by the authors and unedited

Supplementary Figure Legends

**Supplementary Figure 1: (a):** Summary of key metabolic changes in ccRCC comparing to NATs. Abbreviations of metabolites are detailed in **Supplementary Table 4**.

**(b):** Summary of changes in galactose metabolism of ccRCC comparing to NATs in TJ-RCC. Abbreviations of metabolites are detailed in **Supplementary Table 4**.

**Supplementary Figure 2:** Workflow of the processing of snRNA and snATAC data of TJ-RCC

**Supplementary Figure 3: (a):** Heatmap showing peak-to-gene links in malignant cells. Peak-to-gene links represented correlations between peak accessibility in snATAC data and gene expression in paired snRNA data**;**

**(b):** UMAP of snATAC-data of cancer cells colored by predicted gene scores (left) and mRNA expression level of *LRP2* (right). The gene scores were predicted from the snATAC data;

**(c):** Genome accessibility track visualization of *LRP2* with peak peak-to-gene links. Genes translate from 5' to 3' are colored in red while others are colored in blue;

**Supplementary Figure 4:** Smoothened heatmaps created by Single-cell Multiomic Enhancer-based Gene regulatory network inference (scMEGA) show gradually changes alongside pseudotime, corresponding to results from Monocle 3.

**Supplementary Figure 5:** Distinct TF modules associated to IM2-like and DCCD features

Supplementary Figure 1

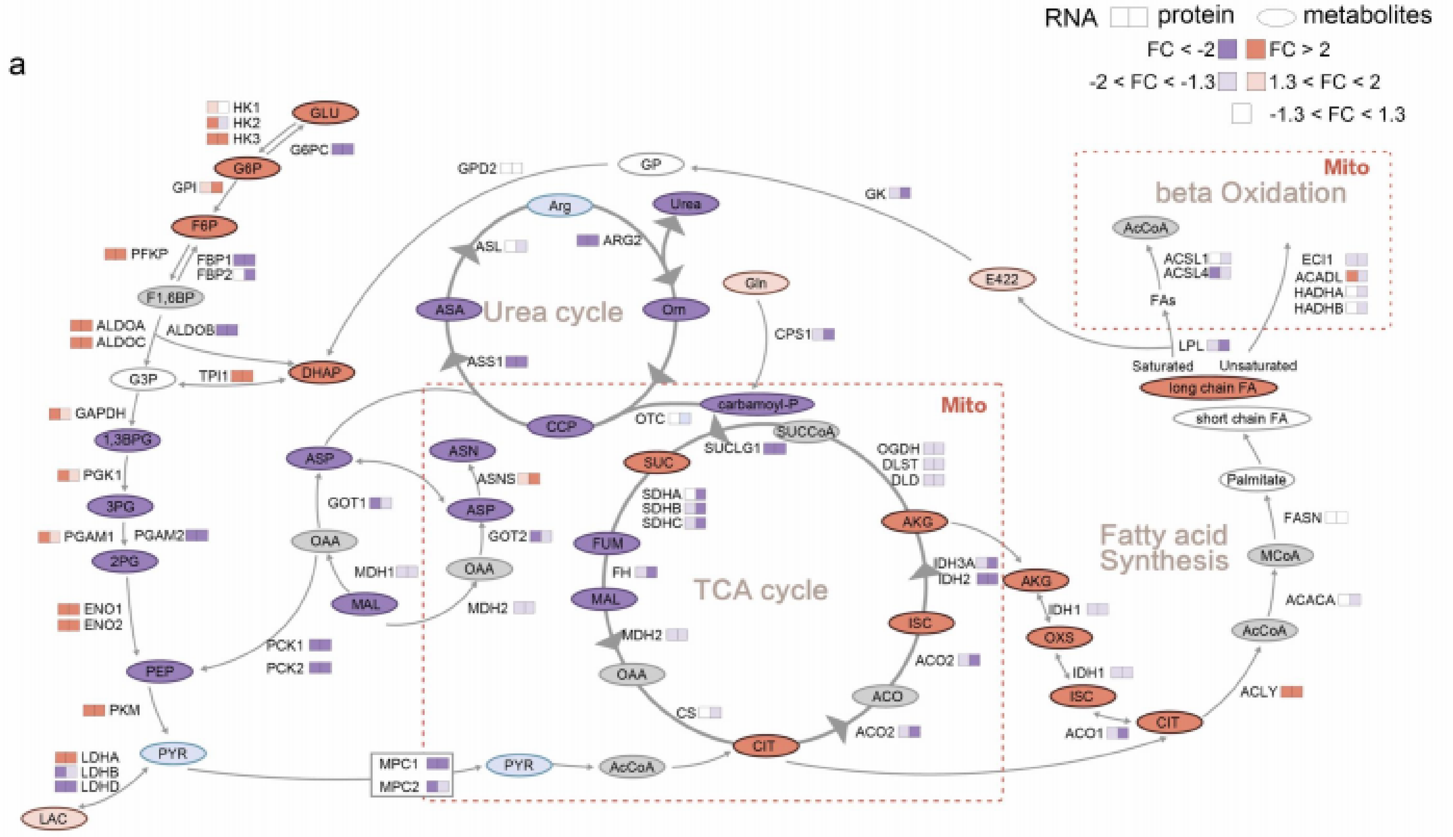

Supplementary Figure 2

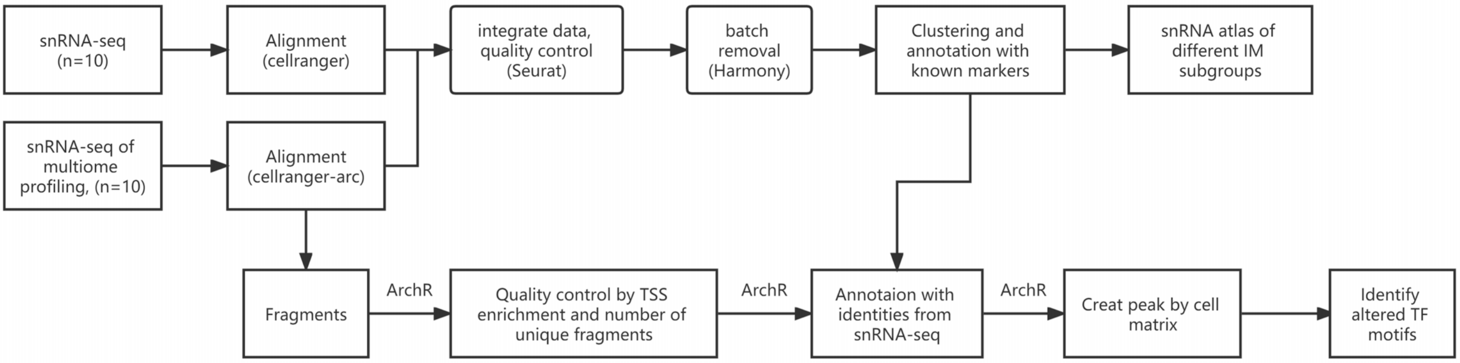

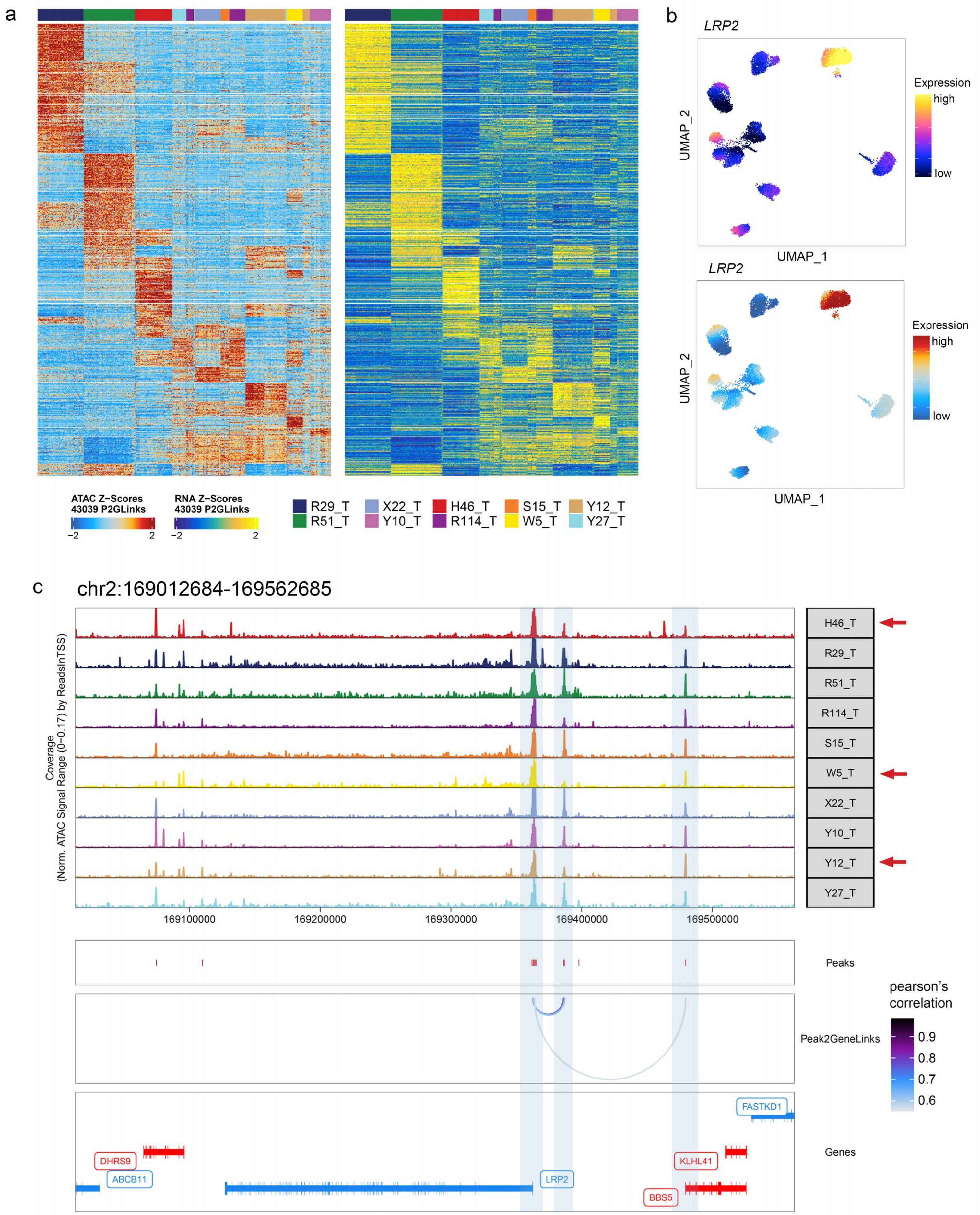
a
ATAC Z−Scores
43039 P2GLinks
−2
2
RNA Z−Scores
43039 P2GLinks
−2
2
R29_T
R51_T
X22_T
Y10_T
H46_T
R114_T
S15_T
W5_T
Y12_T
Y27_T
b
LRP2
UMAP_2
UMAP_1
Expression
high
low
c
chr2:169012684-169562685
Coverage
(Norm. ATAC Signal Range (0-0.17) by ReadsInTSS)
H46_T
R29_T
R51_T
R114_T
S15_T
W5_T
X22_T
Y10_T
Y12_T
Y27_T
169100000
169200000
169300000
169400000
169500000
Peaks
Peak2GeneLinks
pearson's correlation
0.9
0.8
0.7
0.6
Genes
FASTKD1
DHRS9
ABCB11
LRP2
KLHL41
BBS5

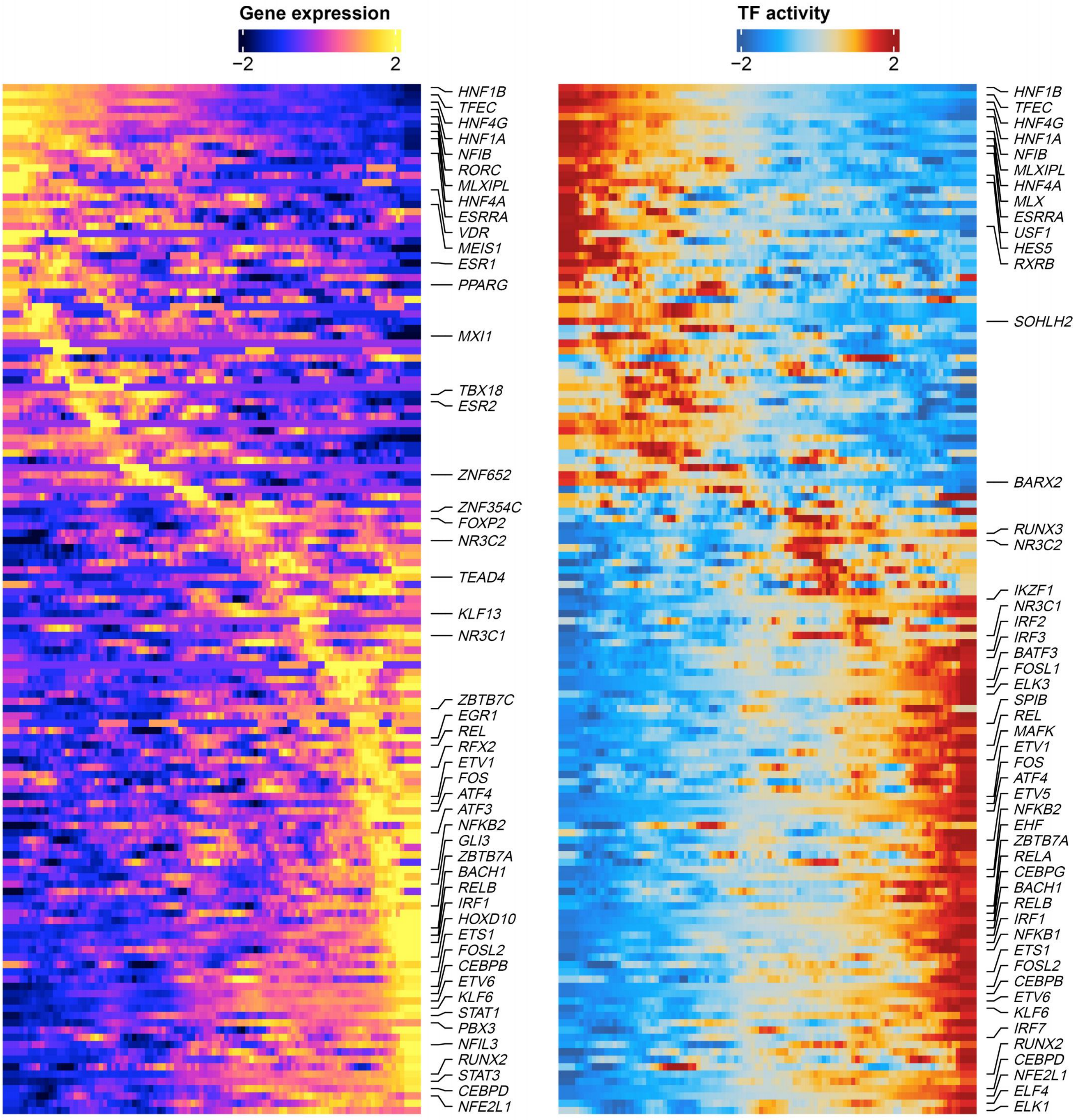
Gene expression
−2
2
HNF1B
TFEC
HNF4G
HNF1A
NFIB
RORC
MLXIPL
HNF4A
ESRRA
VDR
MEIS1
ESR1
PPARG
MXI1
TBX18
ESR2
ZNF652
ZNF354C
FOXP2
NR3C2
TEAD4
KLF13
NR3C1
ZBTB7C
EGR1
REL
RFX2
ETV1
FOS
ATF4
ATF3
NFKB2
GLI3
ZBTB7A
BACH1
RELB
IRF1
HOXD10
ETS1
FOSL2
CEBPB
ETV6
KLF6
STAT1
PBX3
NFIL3
RUNX2
STAT3
CEBPD
NFE2L1
TF activity
−2
2
HNF1B
TFEC
HNF4G
HNF1A
NFIB
MLXIPL
HNF4A
MLX
ESRRA
USF1
HES5
RXRB
SOHLH2
BARX2
RUNX3
NR3C2
IKZF1
NR3C1
IRF2
IRF3
BATF3
FOSL1
ELK3
SPIB
REL
MAFK
ETV1
FOS
ATF4
ETV5
NFKB2
EHF
ZBTB7A
RELA
CEBPG
BACH1
RELB
IRF1
NFKB1
ETS1
FOSL2
CEBPB
ETV6
KLF6
IRF7
RUNX2
CEBPD
NFE2L1
ELF4
ELK1

Supplementary Figure 5

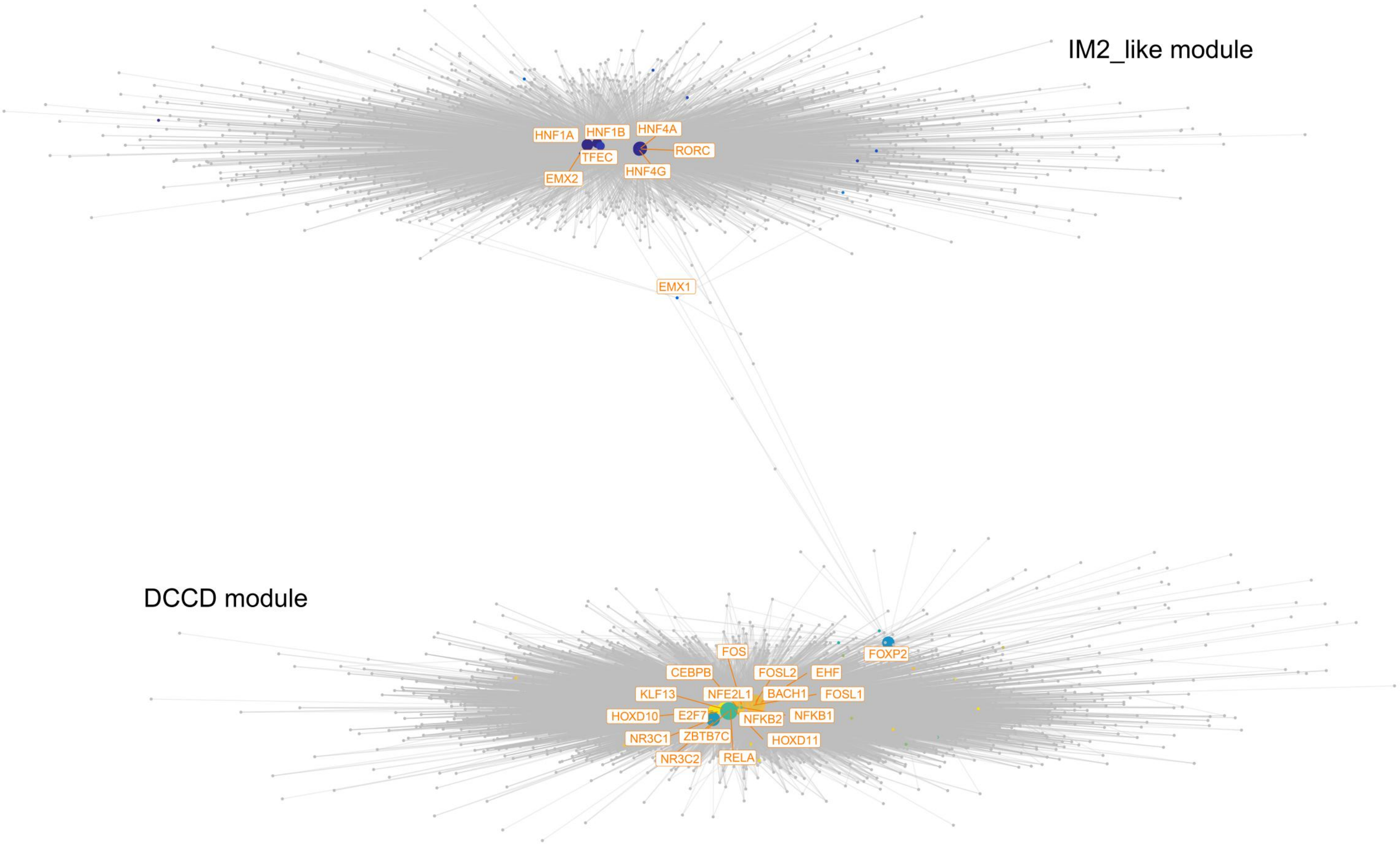

**Supplementary Methods**

**DNA extraction, library preparation and whole exon sequencing (WES)**

In order to minimize discrimination between different omics brought by ITH, we sampled a single site of every primary tumor without visible necrosis. DNA were extracted from tumor and NATs using FastPure Blood/Cell/Tissue/Bacteria DNAIsolation Mini Kit (Vazyme) following the manufacturer's instructions. Genomic DNA extracted from NATs was used as matched benign reference material. Agarose gel electrophoresis were conducted to verify suspected DNA degradation and RNA contamination with 1% agarose gels. Extracted DNA was also quantified with NanoDrop™ One (Thermo, ND-ONE-W). The exome sequences were enriched by Agilent SureSelect Human All Exon V6 system with target region of 58 Mb following the manufacturer's instructions. Subsequently, the Qubit™ dsDNA HS Assay Kit was used with the Qubit™ 3.0 Fluorometer to measure the concentration of dsDNA. The length of DNA segment was verified with D1000 Screen Tape (Agilent). Then, all the libraries underwent paired-end sequencing on a NovaSeq 6000 platform (Illumina) with PE150 strategy.

**RNA extraction, library preparation and whole transcriptome sequencing (WTS)**

Total RNA from 100 tumors and 50 NATs was extracted using the TRIzol™ Reagent (Thermo, 15596018). The 50 NAT samples were sampled from the same specimen as 50 of the tumor samples. RNA quality was verified using a NanoDrop™ One (Thermo, ND-ONE-W). RNA concentration was measured using the Qubit RNA BR (Broad-Range) Assay Kit in a Qubit3.0 Fluorometer (Life Technologies, CA, USA). RNA integrity was assessed using the RNA Nano 6000 Assay Kit with an Agilent Bioanalyzer 4200 (Agilent Technologies, CA, USA). Only RNA samples with RNA integrity Number (RIN) score over 7 were considered high quality samples and subjected to RNA sequencing. Ribo-Zero rRNA Removal Reagent (H/M/R)(illumina USA) was used to remove rRNA from total RNA samples and then Magmetic RNA Beads (Vazyme， China) was used to enrich target mRNA. Sequencing libraries were generated using the NEBNext Ultra Directional RNA Library Prep Kit for Illumina

(NEB, USA) following the manufacturer's instructions. The libraries were then sequenced on an Illumina NovaSeq 6000 platform, and 150 bp paired-end reads were generated.

**Preparation for single nuclei suspension**

20 samples profiled by snRNA-seq or 10X multiome were chosen following randomized strategy. Firstly, we randomly selected 5 samples in each IM subtype. Since only 4 IM4 samples are available after multi-omics sequencing, we finally used 6 IM3 samples and 4 IM4 samples for subsequent profiling. Next, these 20 samples were randomized 1:1 into snRNA-seq group or 10X multiome group. Single nuclei suspension was prepared on ice according to the official standard procedure of 10X Genomics. In summary, frozen tissue samples were chopped with 1ml nuclear lysate (0.1% NP40, 10mM Tris-HCl (pH7.4), 10mM NaCl, 3mM MgCl2, 1mM DTT and 1U/μl RNase inhibitor) and then incubated on ice for 5min. It was filtered into a centrifuge tube with 40 μm cell sifter and then centrifuged at 500g at 4℃ for 5 min. After washing with 1ml PBS+1% BSA+1U/μl RNase inhibitor, it was resuspended with 100μl 0.1× Lysis Buffer (10mM Tris-HCl, 10mM NaCl, 3mM MgCl2, 0.01% NP40, 0.01%Tween-20, 0.001% Digitonin, 1% BSA, 1mM DTT and 1U/μl RNase inhibitor), and then incubated on ice for 2 min. Then we added 1ml Wash Buffer (10mM Tris-HCl, 10mM NaCl, 3mM MgCl2, 1% BSA, 0.1%Tween-20, 1mM DTT and 1U/μl RNase inhibitor), vortexed it well, and then the tube was centrifuged at 500g at 4℃ for 5 min. The supernatant was then discarded. The nuclei were re-suspended with an appropriate amount of Diluted Nuclei Buffer (1×Nuclei Buffer (10X Genomics, PN-2000153), 1mM DTT, 1U/μl RNase inhibitor). Trypan blue dying was then performed to confirm cell death rate per sample. The nuclei suspension passed quality control was then adjusted to a concentration of 4000-8000 nuclei/μl with Diluted Nuclei Buffer. About 20,000 nuclei were used for single-nuclei RNA sequencing (snRNA-seq) or 10×Genomics Multiome sequencing by 10×Genomics Chromium platform.

**Construction of snRNA and snATAC sequencing library**

For single nuclei suspension prepared for snRNA-seq alone, single nuclei suspension was adjusted to a concentration of 700-1200 nuclei/μl with Diluted Nuclei Buffer and then loaded onto a Chromium Next GEM Chip G Single Cell Kit, 48 rxns (10× Genomics, PN-1000120), and processed through the Chromium Controller to generate GEMs (Gel Beads in Emulsion). And then the sequencing libraries were prepared with the Chromium Single Cell 3′ GEM, Library & Gel Bead Kit v3.1, 16 rxns (10× Genomics, PN-1000121) following the manufacturer's instructions. The sequencing libraries were then sequenced by Illumina NovaSeq 6000 platform.

Single nuclei prepared for multiome undergone similar process as above. After generation of GEMs with Chromium Controller on Chromium Next GEM Chip J Single Cell Kit, 48 rxns (10× Genomics, PN-1000230), 10×Genomics Chromium Next GEM Single Cell Multiome ATAC + Gene Expression Reagent Bundle (PN-1000283) was used to prepare the sequencing library according to the manufacturer's instructions. Transcriptome libraries were generated with Dual Index Kit TT Set A, 96 rxns (10× Genomics, PN-1000215) while ATAC-seq libraries were generated with Single Index Kit N Set A, 96 rxns (10× Genomics, PN-1000212). All the libraries were sequenced by Illumina NovaSeq 6000 platform.

**Sample processing and protein extraction**

All the samples were stored in -80℃ refrigerator before processing. 100 tumors and 50 NATs were grinded into powder at low temperature, and then quickly transferred into a liquid nitrogen precooled centrifuge tube. An appropriate amount of PASP protein cracking solution (100 mM ammonium bicarbonate and 8M urea, pH=8) was added into the tube. Then it was shaken and mixed well, and fully lysed by ultrasonic in ice water bath for 5 minutes. After centrifugation at 4℃ and 12000 g for 15 min, supernatant was added with 10 mM DTT and react at 56℃ for 1h. Sufficient IAM was added and then reacted at room temperature in darkness for 1 h. Subsequently, we added 4 quadruple volumes of -20 ℃ precooled acetone into the tube and precipitated at -20℃ for at least 2 h, centrifugated at 4 ℃, 12000g for 15 min, and then collected

the precipitation. After that, 1mL -20°C pre-cooled acetone was added to resuspension the precipitation, and the precipitation was washed and centrifuged at 4°C, 12000g for 15 min. The precipitation was collected and air-dried, and an appropriate amount of protein solution (8M urea, 100mM TEAB, pH=8.5) was added to dissolve the protein precipitation.

The Bradford protein quantification kit was used to verify the quality of protein samples. BSA standard protein solution was prepared according to the manufacturer's instructions, with the concentration gradient ranging from 0 to 0.5μg/μL. BSA standard protein solution with different concentration gradients and the sample solution to be tested with different dilution ratios were added into the 96-well plate, and the volume was filled to 20μL. Each gradient was repeated 3 times. Next, 180μL G250 staining solution was rapidly added into the tube and then placed at room temperature for 5 min to determine the absorbance at 595 nm. The standard curve was drawn with the absorbance of the standard protein solution and it was used to calculate the protein concentration of all the protein samples to be measured. 20 μg protein were extracted from every protein sample to conduct 12% SDS-PAGE gel electrophoresis, with the conditions of 80 V and 20 min for concentrated gel and 120 V and 90 min for separation gel. After electrophoresis, Coomassie bright blue R-250 staining was performed on the gel and decolorized until the strip was clear.

Protein samples were taken and added with DB proteolysis solution (8 M urea, 100 mM TEAB, pH=8.5) to make up the volume to 100 μl. Pancreatic enzyme and 100 mM TEAB buffer were added, mixed and digested at 37°C for 4h. And then pancreatic enzyme and CaCl2 were added to incubate overnight. Then we added formic acid to adjust pH to lower than 3. Then it was centrifuged at room temperature at 12000 g for 5 min. The supernatant was then slowly passed through the C18 desalting column. After that, the column was washed for 3 times with the cleaning fluid (0.1% formic acid, 3% acetonitrile) and then appropriate eluent (0.1% formic acid, 70% acetonitrile) was added. Finally, the filtrate was collected and lyophilized.

**Construction of the DDA spectrum library**

The lyophilized powder was dissolved in liquid A (2% acetonitrile, 98% water, ammonia adjusted to pH=10) and centrifuged at room temperature at 12000 g for 10 min. The chromatography was performed on Waters BEH C18 (4.6×250 mm, 5 μm) column using L-3000 HPLC system with the column temperature set at 45°C. We collected 1 tube per minute and combined them into 4 fractions. Each fraction was lyophilized and dissolved with 0.1% formic acid.

4μg supernatant was taken from each fraction, 0.8μl iRT reagent was added, and then half volume samples were used in the test. Liquid chromatography gradient elution was performed on analytical columns with EASY-nLC 1200 UHPLC (Thermo). Q-exactivetm HF-X mass spectrometer (Thermo) and Nanospray Flex™ (ESI) ion source were used for mass spectrum in Data-dependent acquisition (DDA) mode. The full sweep range of ms was m/z 350-1500. Parent ions with the top 40 ionic strengths in the full scan were selected for fragmentation by high-energy collision cracking (HCD) method and subjected to secondary mass spectrometry detection. The raw data of mass spectrum detection (.raw) is generated for the construction of DDA spectrum library.

**Data Independent Acquisition (DIA) mode protein quantification**

Similar to sample preparation for DDA mode mass spectrum, 4μg supernatant was taken from each sample, 0.8μl iRT reagent was added, and then half of the volume of each sample was used for mass spectrum. Liquid chromatography gradient elution was also performed on analytical columns with EASY-nLC 1200 UHPLC (Thermo). Mass spectrum was performed on Q-exactivetm HF-X mass spectrometer under DIA mode, with the ion spray voltage set at 2.1 kV, the ion transfer tube temperature set at 320°C. The full scanning range of ms was 350-1500 m/z and the resolution of primary MS was set to 60K. After secondary mass spectrum (MS2, variable m/z range, 30K resolution), the original data of mass spectrum detection (.raw) was generated for data analysis.

**Metabolites extraction and LC-MS analysis of tissue samples**

100 tumors and 50 NATs were used to perform metabolomics analysis. For each case, 100 mg frozen tissue was ground in liquid nitrogen and placed into an EP tube. 500μl 80% methanol was added into the tube to extract the metabolites. It was then centrifuged at 4°C, 15000 g for 20 min. The supernatant was then collected and diluted with mass spectrometry grade water until the methanol content is 53%. After centrifuging at 4°C, 15000 g for 15 min, the supernatant was collected for LC-MS.
Mass spectrum was performed with Thermo Scientific™ Q Exactive™ HF. The mass range was from m/z 100 to 1,500. The resolution was set at 70,000 for the full MS scan sand 17,500 for HCD MS/MS scans. The Collision energy was set at 10, 20 and 40eV. The mass spectrometer operated as follows: spray voltage: 3.2kV; sheath gas flow rate: 40 arbitrary units; auxiliary gas flow rate: 10 arbitrary units; capillary temperature: 320°C; Polarity: positive； negative.

**Metabolites extraction and GC-MS analysis of tissue samples**

50 mg tissue per sample was used to conduct GC-MS analysis. 450μl extracting solution (methanol/chloroform volume ratio=3:1) and 10μl L-2-chlorophenylalanine were added and then vortexed for 30s. Subsequently, magnetic beads were added into each EP tube and then grinded at 45 HZ for 5 min, and the whole samples were extracted by ultrasonic for 4 min in ice-water bath. The extract was centrifuged at 4°C, 12000 rpm for 15 min. 300μl of the supernatant per tube was dried in a freeze concentration centrifugal dryer. 60μl mixture of methoxyamine hydrochloride and pyridine (20mg/ml) were added to each sample, and then incubated at 80°C for 30 min. Then 80μl BSTFA (containing 1% TMCS, v/v) was added and incubated at 70°C for 1.5h. When the tube is cooled to the room temperature, 5μl FAMEs was added. Pooled QC samples were prepared by mixing aliquots of all the samples.
GC-MS analysis was performed utilizing an Agilent 7890B gas chromatograph coupled to Pegasus HT (LECO), a time-of-flight mass spectrometer. A DB-5MS capillary column (30 m×250 μm×0.25 μm, Agilent) was used to separate the

derivatives. 1μL aliquot of sample was injected with helium used as the carrier gas in splitless mode. Parameters were set as follows: the front inlet purge flow: 3ml/min; the gas flow rate through the column: 1ml/min. The initial temperature was maintained at 50°C for 1min, and then raised up to 310 °C at a rate of 10°C/min, then kept at 310°C for 8 min. The injection, transfer line, and ion source temperatures were 280, 280and 250°C, respectively. The collision energy was 70eV in electron impact mode. The mass spectrometry data were acquired in full-scan mode with the m/z range from 50 to 500 at a rate of 12.5 spectra per second. The solvent delay time was set to 6.25 min.

## Spatial transcriptomics (ST)

**Tissue preparation, frozen section, fixation, H&E staining and imaging**

12 tumor tissues and 2 NATs used to perform ST were cut into 4-5 $mm^3$ pieces. The blood stains on the tissue surface were wiped with a dust-free paper. Then the tissues were embedded into optimal cutting temperature medium (OCT) and placed at -80°C for rapid freezing. Embedded tissues were then sectioned into 10 μm-thick sections by a Leica microtome (Leica CM 1950, Leica Microsystem, Germany) and then pasted onto the ST microarray (10X Genomics). The sections were dehydrated with isopropyl alcohol for 1 mi and then H&E staining were performed on each section. Bright field images were then taken on a 20× resolution 3D HISTECH Pannoramic MIDI FL full-slide scanner (3DHISTECH LTD.).

**Tissue permeabilization, optimization and sequencing library construction**

Optimization was performed following the manufacturer's instruction (10x Genomics, CG000160). Optimization was performed per sample to confirm the best permeabilization time of each sample. After permeabilization, mRNA released for the cells was captured by primers on the microarray chip. Sequencing libraries were then constructed following Visium Spatial Gene Expression User Guide (10x Genomics, CG000239) and then sequenced by Illumina NovaSeq 6000 platform.

**Sample preparation for spatial metabolomics**

Before being sectioned, the pre-embedded samples were stored at -80 °C. The embedded samples were cut into 10 consecutive sagittal slices about 10 μm-thick by the Leica CM 1950 microtome and then thaw-mounted onto a positive charge desorption plate (Thermo Scientific, U.S.A). Sections were stored at -80 °C before further analysis. Before mass spectrometry imaging (MSI) analysis, these sections were desiccated at -20 °C for 1 h and then at room temperature for 2 h.
