# Supplementary material for: Multi-omic profiling of clear cell renal cell carcinoma identifies metabolic reprogramming associated with disease progression
Source: Nat Genet. 2024 Feb 15;56(3):442–57. doi: 10.1038/s41588-024-01662-5 (PMC10937392; doi:10.1038/s41588-024-01662-5)
Supplement: Supplementary file 2 — Reporting Summary [file 41588_2024_1662_MOESM2_ESM.pdf]

nature portfolio

Corresponding author(s): Ke Chen

Last updated by author(s): Oct 23, 2023

Reporting Summary

Nature Portfolio wishes to improve the reproducibility of the work that we publish. This form provides structure for consistency and transparency in reporting. For further information on Nature Portfolio policies, see our Editorial Policies and the Editorial Policy Checklist.

Statistics

For all statistical analyses, confirm that the following items are present in the figure legend, table legend, main text, or Methods section.

n/a	Confirmed	
☐	☒	The exact sample size (*n*) for each experimental group/condition, given as a discrete number and unit of measurement
☐	☒	A statement on whether measurements were taken from distinct samples or whether the same sample was measured repeatedly
☐	☒	The statistical test(s) used AND whether they are one- or two-sided<br>*Only common tests should be described solely by name; describe more complex techniques in the Methods section.*
☐	☒	A description of all covariates tested
☐	☒	A description of any assumptions or corrections, such as tests of normality and adjustment for multiple comparisons
☐	☒	A full description of the statistical parameters including central tendency (e.g. means) or other basic estimates (e.g. regression coefficient) AND variation (e.g. standard deviation) or associated estimates of uncertainty (e.g. confidence intervals)
☐	☒	For null hypothesis testing, the test statistic (e.g. *F*, *t*, *r*) with confidence intervals, effect sizes, degrees of freedom and *P* value noted<br>*Give P values as exact values whenever suitable.*
☒	☐	For Bayesian analysis, information on the choice of priors and Markov chain Monte Carlo settings
☒	☐	For hierarchical and complex designs, identification of the appropriate level for tests and full reporting of outcomes
☐	☒	Estimates of effect sizes (e.g. Cohen's *d*, Pearson's *r*), indicating how they were calculated

Our web collection on statistics for biologists contains articles on many of the points above.

Software and code

Policy information about availability of computer code

Data collection	No software was used.
Data analysis	Software used include: fastp(v0.20.1),python (v3.7.12), CNVkit (v0.9.10), R (v4.1.2), GISTIC2.0 (v2.0.23), CellRanger sotware (v6.1.2), CellRanger-arc (V2.0.0), SpaceRanger (v2.0.0),Seurat (v4.0.1), sigminer (v2.1.3), edgeR (v3.36.0), ArchR (v1.0.2), ANNOVAR, GATK (v4.2.5.0), sentieon (v202010.04), HISAT2 (v2.1.0), FeatureCounts (v2.0.1), NormalyzerDE (v1.12.0), sva (v3.42.0), Seurat (V4.0.5), Harmony (v0.1.0), COSG (v0.9.0), ComplexHeatmap (v2.16.0), multiOmicsViz (v1.18.0), MACS2 (v2.2.7.1), chromVAR (v1.16.0), SPATA2 (v0.1.0), Monocle (v2.22.0), GSVA (v1.42.0), Monocle 3 (V1.0.0), scMEGA (v0.2.0), clusterProfiler (v4.2.0), ConsensusClusterPlus (v1.58.0), Limma (v3.50.0) and MSiReader (v1.02), scanpy (v1.9.1), destiny (v3.8.1), Proteome Discoverer 2.2 (PD 2.2, Thermo), MS-DIAL, Metaboanalyst 5.0.

For manuscripts utilizing custom algorithms or software that are central to the research but not yet described in published literature, software must be made available to editors and reviewers. We strongly encourage code deposition in a community repository (e.g. GitHub). See the Nature Portfolio guidelines for submitting code & software for further information.

Data

Policy information about availability of data

All manuscripts must include a data availability statement. This statement should provide the following information, where applicable:

- Accession codes, unique identifiers, or web links for publicly available datasets
- A description of any restrictions on data availability
- For clinical datasets or third party data, please ensure that the statement adheres to our policy

Raw sequencing data has been uploaded to the GSA-Human database under accession code PRJCA014547 (https://ngdc.cncb.ac.cn/bioproject/browse/PRJCA014547) but a DAC approval is necessary due to policy restrictions. All the Processed sequencing data have been uploaded to Zenodo (https://zenodo.org/record/8063124). Expression matrix of TCGA KIRC along with clinical features was obtained from UCSC Xena (https://xenabrowser.net/datapages/?cohort=GDC%20TCGA%20Kidney%20Clear%20Cell%20Carcinoma%20(KIRC)&removeHub=https%3A%2F%2Fxena.treehouse.gi.ucsc.edu%3A443). JAVLIN and checkmate datasets were obtained from the supplementary material of the original paper. Data of IMmotion 151 was obtained from EGA database (https://ega-archive.org/studies/EGAS00001004353) with approval from the DAC. Single cell sequencing data of ccRCC was downloaded from Mendeley (http://dx.doi.org/10.17632/nc9bc8dn4m.1). FUSCC referred to the RCC cohort profiled by team from Fudan University Shanghai Cancer Center (FUSCC). PKU referred to the RCC cohort profiled by team from Peking University (PKU). WES data of PKU cohort collected under PRJNA596359 (https://www.ncbi.nlm.nih.gov/sra/?term=PRJNA596359) was downloaded from SRA database. Processed WES data of FUSCC was obtained from NODE (https://www.biosino.org/node) under Project ID: OEP000796. COSMIC database (https://cancer.sanger.ac.uk/cosmic) was used to annotate the SBS signatures in WES data.

Research involving human participants, their data, or biological material

Policy information about studies with human participants or human data. See also policy information about sex, gender (identity/presentation), and sexual orientation and race, ethnicity and racism.

Reporting on sex and gender	This cohort contained males (n = 63) and females (n = 37), corresponding to the sex distribution of ccRCC.
Reporting on race, ethnicity, or other socially relevant groupings	Race, ethnicity, or other socially relevant information was not involved in this study.
Population characteristics	A total of 100 participants, with an age range of 27-84, were included in this study. This cohort contained males (n = 63) and females (n = 37), corresponding to the gender distribution of ccRCC. Baseline population characteristics of patients with ccRCC are detailed in Supplementary Tables 1.
Recruitment	Histopathological diagnosis was confirmed by at least two different pathologists per sample and only ccRCC cases were included in this sequencing cohort. Informed consent was obtained prior to tissue acquisition. Our cohort included treatment-naive ccRCC patients underwent surgery at Wuhan Tongji Hospital in Jul 2020 and Apr 2021 without intentional selection.
Ethics oversight	Institutional Review Board approval (Tongji Hospital) and informed consent was obtained prior to tissue acquisition and analysis.

Note that full information on the approval of the study protocol must also be provided in the manuscript.

Field-specific reporting

Please select the one below that is the best fit for your research. If you are not sure, read the appropriate sections before making your selection.

☒ Life sciences ☐ Behavioural & social sciences ☐ Ecological, evolutionary & environmental sciences

For a reference copy of the document with all sections, see nature.com/documents/nr-reporting-summary-flat.pdf

Life sciences study design

All studies must disclose on these points even when the disclosure is negative.

Sample size	Clinical characteristics are summarized in Table S1. No statistical methods were used to predetermine sample size. 100 tumors with paired adjacent normal tissues (NATs) were used in whole exon sequencing. Sample size of whole transcriptome sequencing, global proteomics, non-target metabolomics were 100 tumors and 50 NATs. The quantification of sample sizes employed in these multi-omics analyses was based on existing norms within the discipline, as established by parallel investigations in the realm of solid tumor multi-omics research (PMID: 33577785, 34534465, 33212010).<br>20 out of 100 tumor samples were selected for single nucleic transcriptome (n=10) or 10X multiome (n=10) sequencing based on molecular subtypes. Furthermore, 10 out of these 20 tumors were randomly selected for spatial transcriptome (n=10) and spatial metabolome (n=10) profiles. A paired normal renal cortex and medulla were sequenced by single nucleic transcriptome, spatial transcriptome and spatial metabolome and was used as a normal control.
Data exclusions	WES data of 5 NATs failed to pass the quality control and no more tissue was available to sequence once again. Hence, paired-tumor samples of these 5 NATs were not involved in SNA and SCNA calling. Instead, we applied GATK germline mutation calling workflow and only select 50

most frequent SNAs occured in the other 95 tumors to stat the total mutation rate.

Replication	The reported findings were replicated across multiple biological samples. Oil red O staining, IHC and immunofluorescenct imaging were performed on 20 different tumor samples and replicated 3 times on each sample. No other experiment was involved.
Randomization	Because all treatment-naive ccRCC patients underwent surgery at Wuhan Tongji Hospital between Jul 2020 and Apr 2021 were involved, acquisition of primary patient tumor samples was not randomized. Samples were randomized by case and control status during RNA isolation or library preparation. Tumor samples involved in single nuclei sequencing and spatial sequencing were randomly selected from 100 cases.
Blinding	Blinding of the tissue was not possible. All analyses were performed in an automated manner across conditions.

Reporting for specific materials, systems and methods

We require information from authors about some types of materials, experimental systems and methods used in many studies. Here, indicate whether each material, system or method listed is relevant to your study. If you are not sure if a list item applies to your research, read the appropriate section before selecting a response.

Materials & experimental systems

n/a	Involved in the study	
☐	☒	Antibodies
☒	☐	Eukaryotic cell lines
☒	☐	Palaeontology and archaeology
☒	☐	Animals and other organisms
☒	☐	Clinical data
☒	☐	Dual use research of concern
☒	☐	Plants

Methods

n/a	Involved in the study	
☒	☐	ChIP-seq
☒	☐	Flow cytometry
☒	☐	MRI-based neuroimaging

Antibodies

Antibodies used

Anti-Human CD8 (1:100, Abcam, Cat# ab178089)
Anti-Human CD31 (1:2000, Proteintech, Cat#:11265-1-AP)
Anti-Human DCN (1:2000, Abcam, Cat# ab277636)
Anti-Human CD3 (1:1000, Proteintech, Cat# 17617-1-AP)
Anti-Human CD163 (1:2000, Proteintech, Cat# 16646-1-AP)
Anti-Human a-SMA/ACTA2 (1:2500, Proteintech, Cat# 14395-1-AP)
Anti-Human PDGFRA (1:500, Abcam, Cat# ab203491)
Anti-Human F13A1 (1:100, Abcam, Cat# ab76105)
Anti-Human ACKR1 (1:200, SAB, Cat# 56458)
Anti-Human CX40 (1:500, BIOSS, Cat# bs-1050R)
Goat Anti-Rabbit IgG H&L (HRP) (1:2000, Abcam, Cat# ab205718)
Goat Anti-Rabbit IgG H&L (Cy3 ®) preadsorbed (1:200, Abcam, Cat# ab6939)
Goat Anti-Rabbit IgG H&L (Alexa Fluor® 488) (1:500, Abcam, Cat# ab150077)
Goat Anti-Rabbit IgG H&L (Alexa Fluor® 594) (1:500, Abcam, Cat# ab150080)
Goat Anti-Rabbit IgG H&L (Cy5 ®) preadsorbed (1:500, Abcam, Cat# ab6564)

Validation

All antibodies used in this study are commercially available. They are validated by the vendors for the specific assay and species used. The validation is available on the vendors website.
Anti-Human CD8: https://www.abcam.cn/products/primary-antibodies/cd8-alpha-antibody-sp239-ab178089.html
Anti-Human CD31: https://www.ptgcn.com/Products/PECAM1-Antibody-11265-1-AP.htm#product-information
Anti-Human DCN: https://www.abcam.cn/products/primary-antibodies/decorin-antibody-epr24097-105-ab277636.html
Anti-Human CD3: https://www.ptgcn.com/products/CD3E-Antibody-17617-1-AP.htm
Anti-Human CD163: https://www.ptgcn.com/products/CD163-Antibody-16646-1-AP.htm
Anti-Human a-SMA/ACTA2: https://www.ptgcn.com/products/ACTA2-Antibody-14395-1-AP.htm
Anti-Human PDGFRA: https://www.abcam.cn/products/primary-antibodies/pdgfr-alpha-antibody-epr22059-270-ab203491.html
Anti-Human F13A1: https://www.abcam.cn/products/primary-antibodies/factor-xiiia-antibody-ep3372-ab76105.html
Anti-Human ACKR1: https://www.sabbiotech.com.cn/g-320124-DARC-Rabbit-mAb-56458.html
Anti-Human CX40: http://www.bioss.com.cn/SpeNew01.asp?id=258&pro37=1&pro33=101&guige01=50ul
Goat Anti-Rabbit IgG H&L (HRP): https://www.abcam.cn/products/secondary-antibodies/goat-rabbit-igg-hl-hrp-ab205718.html
Goat Anti-Rabbit IgG H&L (Cy3 ®) preadsorbed: https://www.abcam.cn/products/secondary-antibodies/goat-rabbit-igg-hl-cy3--preadsorbed-ab6939.html
Goat Anti-Rabbit IgG H&L (Alexa Fluor® 488): https://www.abcam.cn/products/secondary-antibodies/goat-rabbit-igg-hl-alexa-fluor-488-ab150077.html
Goat Anti-Rabbit IgG H&L (Alexa Fluor® 594): https://www.abcam.cn/products/secondary-antibodies/goat-rabbit-igg-hl-alexa-fluor-594-ab150080.html
Goat Anti-Rabbit IgG H&L (Cy5 ®) preadsorbed: https://www.abcam.cn/products/secondary-antibodies/goat-rabbit-igg-hl-cy5--preadsorbed-ab6564.html
Goat Anti-Rabbit IgG H&L (Cy3 ®) preadsorbed: https://www.abcam.cn/products/secondary-antibodies/goat-rabbit-igg-hl-cy3--preadsorbed-ab6939.html
